# Clinical impact of extubation in the operating room after cardiac surgery: a retrospective analysis of a prospective registry

**DOI:** 10.1016/j.bjane.2026.844761

**Published:** 2026-04-24

**Authors:** Patricia Silva de Marco, Pedro Henrique Romani Lauand, Luiz Carlos Volpi Júnior, Raphael Fernandes Cardoso Vianna Alves, Maria Paula Belini, Lilia Nigro Maia, Maurício Nassau Machado, Marcelo Arruda Nakazone

**Affiliations:** Faculdade de Medicina de São José do Rio Preto (FAMERP), Departamento de Cardiologia e Cirurgia Cardiovascular, São José do Rio Preto, SP, Brazil

**Keywords:** Airway extubation, Cardiac surgical procedures, Coronary artery bypass, Propensity score, Acute kidney injury, Mortality

## Abstract

**Background:**

The practice of immediate extubation in the Operating Room (OR) after cardiac surgery involving cardiopulmonary bypass remains debated. Concerns persist regarding its safety profile compared to conventional fast-track weaning in the Intensive Care Unit (ICU).

**Methods:**

This retrospective analysis of a prospective single-center registry included 846 adult patients undergoing isolated Coronary Artery Bypass Grafting (CABG) or Heart Valve Surgery (HVS). To rigorously account for indication bias, we employed a doubly robust estimation approach, combining Stabilized Inverse Probability of Treatment Weighting (SIPTW) with multivariable regression adjustment for residual confounding.

**Results:**

Of the 846 patients, 115 (13.6%) were extubated in the OR and 731 (86.4%) in the ICU. The weighted analysis successfully balanced baseline characteristics. Extubation in the OR was associated with a significant reduction in 30-day mortality (adjusted Hazard Ratio [aHR = 0.16]; 95% CI 0.03–0.92; p = 0.040) and acute kidney injury (adjusted Odds Ratio [aOR = 0.55]; 95% CI 0.31–0.99; p = 0.046). Additionally, it reduced the likelihood of prolonged ICU stay (> 14 days) (aOR = 0.10; p = 0.032). Contrary to unadjusted analyses, there was no significant association with respiratory tract infection (p = 0.163), reintubation (p = 0.103), or other adverse events.

**Conclusion:**

In this single-center, robustly adjusted analysis, extubation in the OR was associated with favorable outcomes, including lower 30-day mortality and reduced resource utilization in highly selected patients. Previous concerns regarding increased reintubation rates were not substantiated.

## Introduction

Advances in anesthetic, surgical and Cardiopulmonary Bypass (CPB) techniques have significantly reduced duration of Mechanical Ventilation (MV) and clinical complications after cardiac surgeries.^1^^,^^2^

Early extubation (typically defined as within 6 hours of Intensive Care Unit [ICU] arrival) is now a well-established quality metric that optimizes resource utilization and promotes faster recovery.^3^^,^^4^ However, the practice of immediate extubation in the Operating Room (OR) remains a subject of debate.

While OR extubation has been demonstrated to be feasible in selected low-risk populations, such as those undergoing Off-Pump Coronary Artery Bypass Grafting (OPCABG) or minimally invasive procedures,^5^, ^6^, ^7^, ^8^ its safety and efficacy in a broader spectrum of cardiac surgery patients requiring CPB are less established. Proponents argue that avoiding ICU ventilation minimizes exposure to positive pressure ventilation and sedation, potentially reducing length of stay and costs.^9^^,^^10^ Conversely, critics caution that immediate extubation may mask early postoperative instability, potentially increasing the risk of respiratory failure, reintubation, and bleeding complications.^11^^,^^12^ Recent observational studies using propensity-score matching have yielded conflicting results, with some reporting safety and others suggesting increased hazards.^12^

A critical limitation of prior studies is the potential for indication bias, where healthier patients are preferentially selected for OR extubation, confounding the assessment of true clinical impact. To address this gap, we conducted a retrospective analysis of a prospectively collected registry using a doubly robust statistical approach.

The primary objective of this study was to evaluate the association between OR extubation and 30-day all-cause mortality in adult patients undergoing isolated Coronary Artery Bypass Grafting (CABG) or Heart Valve Surgery (HVS). Our prespecified primary hypothesis was that extubation in the OR is a feasible approach associated with satisfactory clinical results and reduced resource utilization when compared to conventional ICU extubation, after rigorous adjustment for baseline risk and operative characteristics.

## Methods

### Patients

This single-center study was designed as a retrospective analysis of data from a prospectively maintained registry. We evaluated all adult patients (≥ 18 years) who underwent isolated CABG or HVS in a tertiary hospital between September 2018 and August 2023 (60 months). Patients who underwent aortic surgeries, combined procedures, congenital heart disease correction, or heart transplantation were excluded. The study was conducted in accordance with the Declaration of Helsinki and approved by the local research ethics committee (CAAE: 81704424.0.0000.5415), which waived the need for individual informed consent due to the retrospective nature of the analysis.

Demographic and laboratory data were obtained from electronic medical records. The risk of postoperative death was assessed using the InsCor score, a locally validated risk model that includes 10 variables, each one with a different score: age > 70 years, female sex, CABG combined with HVS, myocardial infarction < 90 days, reoperation, aortic valve surgery, tricuspid valve surgery, baseline creatinine level > 2 mg.dL^−1^, left ventricular ejection fraction < 30% and critical preoperative state.^13^

The primary outcome was all-cause mortality within 30 days. Secondary outcomes included Acute Kidney Injury (AKI), defined according to the Kidney Disease: Improving Global Outcomes (KDIGO) criteria,^14^ respiratory tract infection, reintubation, ICU readmission, and prolonged ICU length of stay (> 14 days). The outcome “mechanical ventilation > 24 hours” was excluded from the comparative analysis to avoid structural bias relative to the intervention.

### Postoperative management

Postoperative care followed a standardized institutional protocol applied uniformly to all patients, regardless of the extubation location. For patients admitted to the ICU requiring mechanical ventilation, sedation was typically maintained with propofol or dexmedetomidine and titrated to achieve a light sedation target (Richmond Agitation-Sedation Scale [RASS] -1 to 0) to facilitate neurological assessment and weaning. Patients extubated in the OR received supplemental oxygen and standard non-invasive monitoring. The decision of extubation in the OR was made by consensus of the attending anesthesiologist and surgeon based on real-time clinical assessment, reflecting routine practice rather than a predefined protocol. Analgesia for both groups was managed using a multimodal approach, including intravenous dipyrone and opioids as needed to maintain a Visual Analog Scale (VAS) score < 4. A dedicated physiotherapy team initiated respiratory rehabilitation and early mobilization protocols on the first postoperative day, contingent on hemodynamic stability. This standardized pathway ensured that differences in outcomes were not attributable to variations in post-extubation nursing or physiotherapy care.

### Missing data handling

The dataset was complete for all baseline covariates included in the propensity score model and outcome variables. Specifically, there were no missing values for age, sex, comorbidities, renal function, or operative times across the 846 patients included in the final analysis. Therefore, complete-case analysis was performed without the need for imputation methods.

### Statistical analysis

Continuous variables were assessed for normality using the Shapiro-Wilk test and visual inspection of histograms. To ensure uniformity in data presentation, all continuous variables are reported as median (Interquartile Range, IQR) and were compared using the Mann-Whitney *U*-test. Categorical variables are presented as absolute numbers (n) and percentages (%) and compared using the Chi-Square test or Fisher’s exact test. To address potential confounding by indication and baseline differences between the groups (OR extubation vs. ICU extubation), we employed a doubly robust estimation approach utilizing Stabilized Inverse Probability of Treatment Weighting (SIPTW). A propensity score for the likelihood of being extubated in the OR was calculated using a multivariable logistic regression model including the following covariates: age (years), sex (reference: male), Chronic Obstructive Pulmonary Disease (COPD), hypertension, diabetes mellitus, baseline estimated Glomerular Filtration Rate (eGFR), calculated using the Chronic Kidney Disease Epidemiology Collaboration (CKD-EPI) 2021 equation,^15^ left ventricular function (reference: Left Ventricular Ejection Fraction [LVEF] ≥ 50%), urgent/emergent surgery, type of surgery (reference: CABG), on-pump status, and duration of CPB (minutes). Stabilized weights were calculated to minimize the influence of extreme weights. Covariate balance after weighting was assessed using Standardized Mean Differences (SMD), with an SMD < 0.10 considered indicative of negligible imbalance (Supplementary Table S1).

Outcome analyses were performed on the weighted population using the Complex Samples module of IBM SPSS Statistics v.27 (IBM Corp, Armonk, NY). For the primary outcome (30-day mortality), a weighted Cox proportional hazards regression model was used. For secondary outcomes, weighted binary logistic regression models were applied. To ensure robustness against residual confounding, particularly for age, all baseline covariates used in the propensity score were also included as adjusters in the final outcome models (doubly robust adjustment). Results are presented as adjusted Hazard Ratios (aHR) or adjusted Odds Ratios (aOR) with 95% Confidence Intervals (95% CI). A two-tailed p-value < 0.05 was considered statistically significant.

## Results

### Study population and propensity score weighting

**Figure 1:**
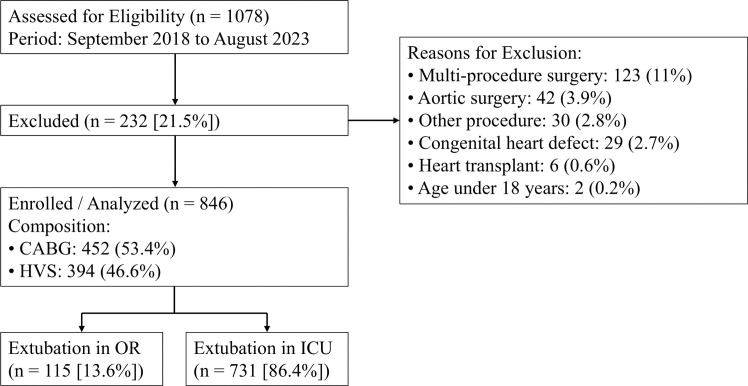
Flow diagram of the study population selection process. Note: CABG, Coronary Artery Bypass Grafting; HVS, Heart Valve Surgery; OR, Operating Room; ICU, Intensive Care Unit. Values are presented as number (n) and percentage (%).

A total of 1,078 patients were assessed for eligibility. After excluding 232 patients (21.5%) primarily due to multi-procedure or aortic surgeries, the final study population comprised 846 patients (Fig. 1). Of these, 115 (13.6%) were extubated in the OR and 731 (86.4%) in the ICU. In the original unadjusted cohort, patients extubated in the OR were significantly younger (median 60 vs 63 years, p = 0.020), had a lower prevalence of hypertension (66.1% vs 74.8%, p = 0.048), better baseline renal function (median eGFR 77 vs. 69 mL.min^−1^/1.73 m^2^, p = 0.001), and shorter duration of CPB (median 80 vs. 89 min, p < 0.001) compared to the ICU group (Table 1).

**Table 1:** Baseline demographic and clinical characteristics of the study population before and after Stabilized Inverse Probability of Treatment Weighting (SIPTW).

		Unadjusted Cohort			Weighted Cohort (SIPTW)		
	Overall	OR extubation	ICU extubation	p-value	OR extubation	ICU extubation	p-value
	n = 846	n = 115	n = 731		n = 108	n = 735	
**Demographics & Risk**							
Age (years)	62 (55‒69)	60 (51‒67)	63 (56‒70)	0.02	60 (51‒67)	62 (55‒70)	0.024
Male sex	578 (68.3)	73 (63.5)	505 (69.1)	0.23	(66.0)	(67.6)	0.704
Body mass index (kg.m^−2^)	27 (25‒31)	27 (25‒31)	27 (24‒30)	0.434	28 (26‒31)	27 (25‒30)	0.136
InsCor	3 (2‒5)	2 (2‒5)	4 (2‒7)	< 0.001	2 (2‒4)	4 (2‒6)	< 0.001
**Comorbidities**							
Chronic obstructive pulmonary disease	39 (4.6)	4 (3.5)	35 (4.8)	0.534	(2.7)	(4.3)	0.608
Hypertension	623 (73.6)	76 (66.1)	547 (74.8)	0.048	(74.7)	(73.7)	0.781
Diabetes mellitus	298 (35.2)	39 (33.9)	259 (35.4)	0.751	(36.0)	(34.9)	0.794
**Cardiac & Renal Status**							
**Left ventricular ejection fraction**							
> 50%	632 (74.7)	94 (81.7)	538 (73.6)	0.062	(73.2)	(75.3)	0.64
31% to 50%	164 (19.4)	18 (15.7)	146 (20.0)	0.276	(24.1)	(18.8)	0.196
≤ 30%	50 (5.9)	3 (2.6)	47 (6.4)	0.106	(2.7)	(5.9)	0.188
Baseline SCr (mg.dL^−1^)	1.10 (0.90‒1.34)	1.10 (0.90‒1.20)	1.10 (0.91‒1.40)	0.003	1.10 (0.99‒1.27)	1.10 (0.90‒1.36)	0.984
Baseline e-GFR (CKD-EPI 2021; mL.min^−1^/1.73 m^2^)	70 (54‒87)	77 (61‒91)	69 (53‒86)	0.001	71 (54‒84)	70 (54‒88)	0.726
**Operative data**							
**Surgery incidence**							
Previous cardiac surgery	78 (9.2)	7 (6.1)	71 (9.7)	0.212	(1.3)	(10.6)	0.001
**Surgical Priority**							
Urgent/Emergent surgery	350 (41.4)	32 (27.8)	318 (43.5)	0.002	(43.2)	(40.7)	0.576
Isolated CABG	452 (53.4)	43 (37.4)	409 (56.0)	< 0.001	(53.2)	(52.9)	0.956
On-pump surgery	826 (97.6)	99 (86.1)	727 (99.5)	< 0.001	(97.6)	(97.3)	1,000
CPB (min)	88 (75‒102)	80 (68‒95)	89 (76‒104)	< 0.001	91 (74‒101)	87 (74‒102)	0.779

Footnotes: Values are presented as n (%) for categorical data or as median (IQR) for continuous variables. SIPTW, Stabilized Inverse Probability of Treatment Weighting; OR, Operating Room; ICU, Intensive Care Unit; SCr, Serum Creatinine; eGFR, Estimated Glomerular Filtration Rate (CKD-EPI); CABG, Coronary Artery Bypass Grafting; CPB, Cardiopulmonary Bypass.

*The p-values for the weighted cohort are derived from Complex Samples General Linear Models. Note: The weighted sample size (n = 843) represents the sum of stabilized weights and differs slightly from the original sample size (n = 846) due to statistical adjustments.

To account for baseline imbalances and potential indication bias, SIPTW was applied. The weighting procedure successfully balanced the covariates between groups, reducing the SMD to < 0.10 for critical variables, including renal function and CPB duration (Supplementary Table S1). A slight residual imbalance remained for age, which was subsequently adjusted for using a doubly robust approach in the final outcome models. The weighted sample size (n = 843) represents the sum of stabilized weights and differs slightly from the original sample size (n = 846) due to statistical adjustments; no patients were excluded from the analysis. The baseline characteristics for both the unadjusted and weighted cohorts are presented in Table 1.

### Primary outcome: 30-day mortality

The unadjusted intra-hospital mortality rate within 30 days was 1.7% (2/115) in the OR extubation group versus 6.6% (48/731) in the ICU extubation group (p = 0.041) (Table 2). In an exploratory unadjusted analysis, non-survivors were significantly older, had worse renal function, and longer CPB times compared to survivors (Supplementary Tables S2–S6). After applying SIPTW and adjusting for all baseline covariates (doubly robust Cox regression), extubation in the OR was associated with a significant reduction in the risk of 30-day all-cause mortality (aHR = 0.16; 95% CI 0.03 to 0.92; p = 0.040) (Table 3).

**Table 2:** Unadjusted clinical complications, ICU stay, and mortality of patients undergoing cardiac surgery according to extubation location.

	Overall	OR extubation	ICU extubation	p-value
	n = 846	n = 115	n = 731	
**Postoperative outcomes**				
Acute kidney injury (KDIGO)^a^	446 (52.7)	46 (40.0)	400 (54.7)	0.003
KDIGO 1	373 (44.1)	43 (37.4)	330 (45.1)	0.120
KDIGO 2	36 (4.3)	3 (2.6)	33 (4.5)	0.460
KDIGO 3	37 (4.4)	0 (0.0)	37 (5.1)	0.014
RRT up to 7 days	14 (1.7)	0 (0.0)	14 (1.9)	0.237
Reoperation for bleeding/tamponade	24 (2.8)	1 (0.9)	23 (3.1)	0.234
Acute atrial fibrillation	137 (16.2)	17 (14.8)	120 (16.4)	0.659
Respiratory infection	188 (22.2)	14 (12.2)	174 (23.8)	0.005
Tracheal reintubation up to 7 days	32 (3.8)	2 (1.7)	30 (4.1)	0.296
Deep sternal wound infection	37 (4.4)	3 (2.6)	34 (4.7)	0.319
Type 1 neurological injury	36 (4.3)	4 (3.5)	32 (4.4)	0.807
**Discharge and mortality**				
CS-ICU readmission	48 (5.7)	4 (3.5)	44 (6.0)	0.274
CS-ICU LOS up to 30 days	4 (3 - 6)	3 (2 - 4)	4 (3 - 6)	< 0.001
Long LOS (> 14 days)	48 (5.7)	1 (0.9)	47 (6.4)	0.017
**30-day mortality**	50 (5.9)	2 (1.7)	48 (6.6)	0.041

Footnotes: Values are presented as n (%) for categorical variables and median (IQR) for continuous variables. The p-values calculated using Chi-Square test, Fisher’s exact test, or Mann-Whitney *U*-test, as appropriate. These represent unadjusted comparisons.

a Acute Kidney Injury defined according to KDIGO (Kidney Disease: Improving Global Outcomes definition and staging) criteria.

**Table 3:** Independent predictors of 30-day mortality and postoperative complications: doubly robust analysis.

Outcome	Adjusted Effect Size^a^ (95% CI)	p-value
Primary Outcome		
30-Day Mortality (aHR)	0.16 (0.03 – 0.92)	0.040
Secondary Outcomes (Renal & Utilization)		
Acute Kidney Injury (aOR)	0.55 (0.31 – 0.99)	0.046
Prolonged ICU Stay > 14 days (aOR)	0.10 (0.01 – 0.82)	0.032
ICU Readmission (aOR)	0.37 (0.11 – 1.31)	0.124
Secondary Outcomes (Safety)		
Respiratory Tract Infection (aOR)	0.59 (0.28 – 1.24)	0.163
Reintubation < 7 days (aOR)^a^	0.26 (0.05 – 1.31)	0.103
Atrial Fibrillation (aOR)	1.11 (0.58 – 2.14)	0.758

Footnotes: Values represent adjusted Hazard Ratio (aHR) for mortality (Cox Regression) and adjusted Odds Ratio (aOR) for secondary outcomes (Logistic Regression). All models were weighted by SIPTW (Stabilized Inverse Probability of Treatment Weighting) and further adjusted for the following covariates: Age, Sex, COPD, Hypertension, Diabetes, Baseline eGFR, Left Ventricular Function, Urgency of Surgery, Type of Surgery (CABG vs. Valve), On-pump status, and CPB duration. Doubly robust estimation combines SIPTW with regression adjustment for the same covariates, providing consistent estimates if either the propensity score or outcome model is correctly specified.

a Due to the low number of reintubation events (n = 32), estimates should be interpreted with caution. CI, Confidence Interval.

### Subgroup analysis

In the subgroup of patients undergoing isolated CABG (n = 446), no deaths occurred in the OR extubation arm (0/58, 0.0%), preventing the calculation of a specific hazard ratio due to perfect separation of data. In the HVS subgroup (n = 397), mortality was 3.9% (2/51) in the OR group versus 8.4% (29/346) in the ICU group. While a formal statistical test of interaction between extubation location and surgery type was limited by the absence of events in the CABG-OR arm, the direction of benefit appeared consistent across procedural types, with no mortality events observed in the revascularization group, although the small sample size precludes definitive safety conclusions (Table S7‒S8).

### Secondary outcomes

Results of the doubly robust logistic regression analyses for secondary outcomes are presented in Table 3. OR extubation was associated with a significant reduction in the odds of developing AKI (KDIGO stage 1‒3) (aOR = 0.55; 95% CI 0.31 to 0.99; p = 0.046) and a significant reduction in the likelihood of prolonged ICU stay (> 14 days) (aOR = 0.10; 95% CI 0.01 to 0.82; p = 0.032).

Regarding safety outcomes, there was no statistically significant association between OR extubation and the risk of reintubation within 7 days (aOR = 0.26; 95% CI 0.05 to 1.31; p = 0.103), postoperative atrial fibrillation (aOR = 1.11; 95% CI 0.58 to 2.14; p = 0.758), or ICU readmission (aOR = 0.37; 95% CI 0.11 to 1.31; p = 0.124). Unlike the unadjusted analysis, the robust adjusted model showed no statistically significant difference in the risk of respiratory tract infection (aOR = 0.59; 95% CI 0.28 to 1.24; p = 0.163), suggesting that previous unadjusted differences were likely driven by baseline patient characteristics (Table 3).

## Discussion

This study evaluated the impact of OR extubation on clinical outcomes and 30-day mortality in 846 patients undergoing isolated CABG or HVS. Using a doubly robust analytical approach that combined SIPTW with covariate adjustment, our findings suggest that extubation in the OR may be associated with a significant reduction in 30-day all-cause mortality, AKI, and prolonged ICU length of stay. Conversely, after rigorous adjustment for baseline differences, we found no significant association between OR extubation and respiratory tract infection, reintubation rates, or other adverse events, suggesting that the apparent protective effect against infection observed in our unadjusted analysis ‒ and potentially in prior observational research ‒ was likely driven by baseline selection bias rather than the intervention itself.

Our primary finding ‒ a significant association with reduced 30-day mortality (aHR = 0.16; 95% CI 0.03–0.92; p = 0.040) ‒ is Noteworthy. Although a doubly robust adjustment was employed to mitigate selection bias, the observed magnitude of effect may partly reflect that successful OR extubation acts as a surrogate marker for optimal intraoperative physiology and surgical performance. Nevertheless, the potential physiological benefits of avoiding prolonged positive pressure ventilation cannot be disregarded as a contributing factor to these favorable outcomes.^16^ Notably, in our subgroup analysis of isolated CABG patients, no deaths occurred in the OR extubation group (0/58). While this finding points toward the feasibility of the strategy in revascularization procedures, the small sample size and absence of events preclude definitive safety conclusions. Although formal statistical testing for interaction was limited by this “zero-event” rate in the intervention arm, the direction of benefit appeared consistent across procedure types.

A critical contribution of our study is the refutation of recent safety concerns. A large propensity-matched analysis by Hawkins et al.^12^ suggested that OR extubation was associated with increased rates of reintubation and reoperation for bleeding. In contrast, our doubly robust analysis found no significant association with reintubation (aOR = 0.26; 95% CI 0.05–1.30; p = 0.103) (Table 3). Furthermore, we observed no signal of increased risk for adverse bleeding events requiring reoperation (0.9% vs. 3.1%, p = 0.234) (Table 2), differing from the findings of Hawkins et al.^12^ Our findings align more closely with James et al.^3^ and e Silva et al.,^9^ who reported that immediate extubation does not compromise safety when performed in selected patients. The absence of increased bleeding or reintubation in our cohort likely reflects the careful intraoperative selection criteria (hemodynamic stability, normothermia, and hemostasis) used by our team, supporting the feasibility of this approach in tertiary centers.

A key finding of our doubly robust analysis is that the protective effect against respiratory infections observed in the unadjusted data (Table 2) was no longer statistically significant after adjustment (aOR = 0.59; 95% CI 0.28–1.24; p = 0.163) (Table 3). In our initial univariable comparison, OR extubation appeared to significantly reduce the risk of infection. However, the robust SIPTW adjustment revealed that this association was likely influenced by the healthier baseline profile of patients selected for OR extubation (younger, better renal function). This result is methodologically reassuring and consistent with the randomized trial by Nicholson et al.,^17^ which found no difference in pulmonary function between early and delayed extubation. This underscores the importance of using advanced statistical methods to disentangle true clinical effects from baseline characteristics.^18^

Consistent with the literature on fast-track protocols,^4^^,^^10^ OR extubation was strongly associated with reduced resource utilization, specifically a 90% reduction in the odds of prolonged ICU stay (> 14 days). Furthermore, we observed a reduction in AKI (aOR = 0.55; 95% CI 0.31–0.99; p = 0.046), which persisted even after adjusting for CPB duration and baseline function (Table 3). This suggests a potential hypothesis that the preservation of spontaneous ventilation and avoidance of positive pressure might contribute to improved renal perfusion, a hypothesis supported by prior physiological studies.^6^ While our findings are consistent with this physiological rationale, further prospective studies are required to confirm this mechanism.

### Limitations

Our study has limitations inherent to its retrospective design. Despite robust statistical adjustment using SIPTW and doubly robust estimation, residual confounding by unmeasured variables (e.g., intraoperative ease of separation from bypass, specific surgeon preference) cannot be completely excluded. Second, the specific subgroup analysis for isolated CABG was limited by the absence of mortality events in the OR extubation arm, preventing the calculation of specific hazard ratios for that subset. Finally, the decision to extubate was based on clinical consensus rather than a rigid protocol, which introduces variability but also reflects real-world clinical judgement. We also acknowledge that urine output criteria for AKI were not available for all patients, limiting our definition to serum creatinine changes. Furthermore, although our weighting approach balanced patient acuity, the study period (2018–2023) encompassed the COVID-19 pandemic and potential variations in practice over time. Specifically, the COVID-19 pandemic period (2020‒2021) may have affected patient selection, surgical volume, and postoperative management protocols, introducing a possibility of temporal confounding that could not be fully adjusted for. Finally, we acknowledge that the primary outcome of 30-day mortality was based on a low number of events in the OR extubation group. Consequently, the mortality estimates and its associated adjusted hazard ratio (95% CI 0.03–0.92) should be interpreted with caution due to the uncertainty inherent in small event counts and the resulting wide confidence interval.

## Conclusion

This study indicates that, in a highly selected population, extubation in the OR is a safe strategy associated with reduced 30-day mortality, lower risk of AKI, and shorter ICU stay in selected patients undergoing cardiac surgery. Concerns regarding increased reintubation or bleeding were not substantiated in this analysis. These findings from a tertiary center support the feasibility of OR extubation for carefully selected candidates.

## Data availability statement

The datasets generated and/or analyzed during the current study are available from the corresponding author upon reasonable request.

## Declaration of Generative AI and AI-assisted technologies in the writing process

During the preparation of this work the author(s) used Gemini, a large language model from Google, in order to improve language and readability, assist in structuring the manuscript sections (Abstract, Introduction, Methods, Results, and Discussion), summarize statistical results into text, and refine the overall narrative clarity. After using this tool, the author(s) reviewed and edited the content as needed and take full responsibility for the content of the publication.

## Authors' contributions

Patricia Silva de Marco: Substantial contributions to the conception of the work; drafting the work and critical review of important intellectual content; acquisition and analysis of data for the study; consent to be responsible for all aspects of the work, ensuring that issues related to the accuracy or integrity of any part of the work are properly investigated and resolved.

Pedro Henrique Romani Lauand: Substantial contributions to the conception of the work; acquisition and analysis of data for the study.

Luiz Carlos Volpi Júnior: Substantial contributions to the conception of the work; acquisition and analysis of data for the study.

Raphael Fernandes Cardoso Vianna Alves: Substantial contributions to the conception of the work; acquisition and analysis of data for the study.

Maria Paula Belini: Substantial contributions to the conception of the work; acquisition and analysis of data for the study.

Lilia Nigro Maia: Substantial contributions to the conception of the work; critical review of important intellectual content.

Maurício Nassau Machado: Substantial contributions to the conception of the work; drafting the work and critical review of important intellectual content; acquisition and analysis of data for the study; critical review of important intellectual content; final approval of the version to be published.

Marcelo Arruda Nakazone: Substantial contributions to the conception of the work; Drafting the work and critical review of important intellectual content; Acquisition and analysis of data for the study; Critical review of important intellectual content; Final approval of the version to be published.

**Figure:**
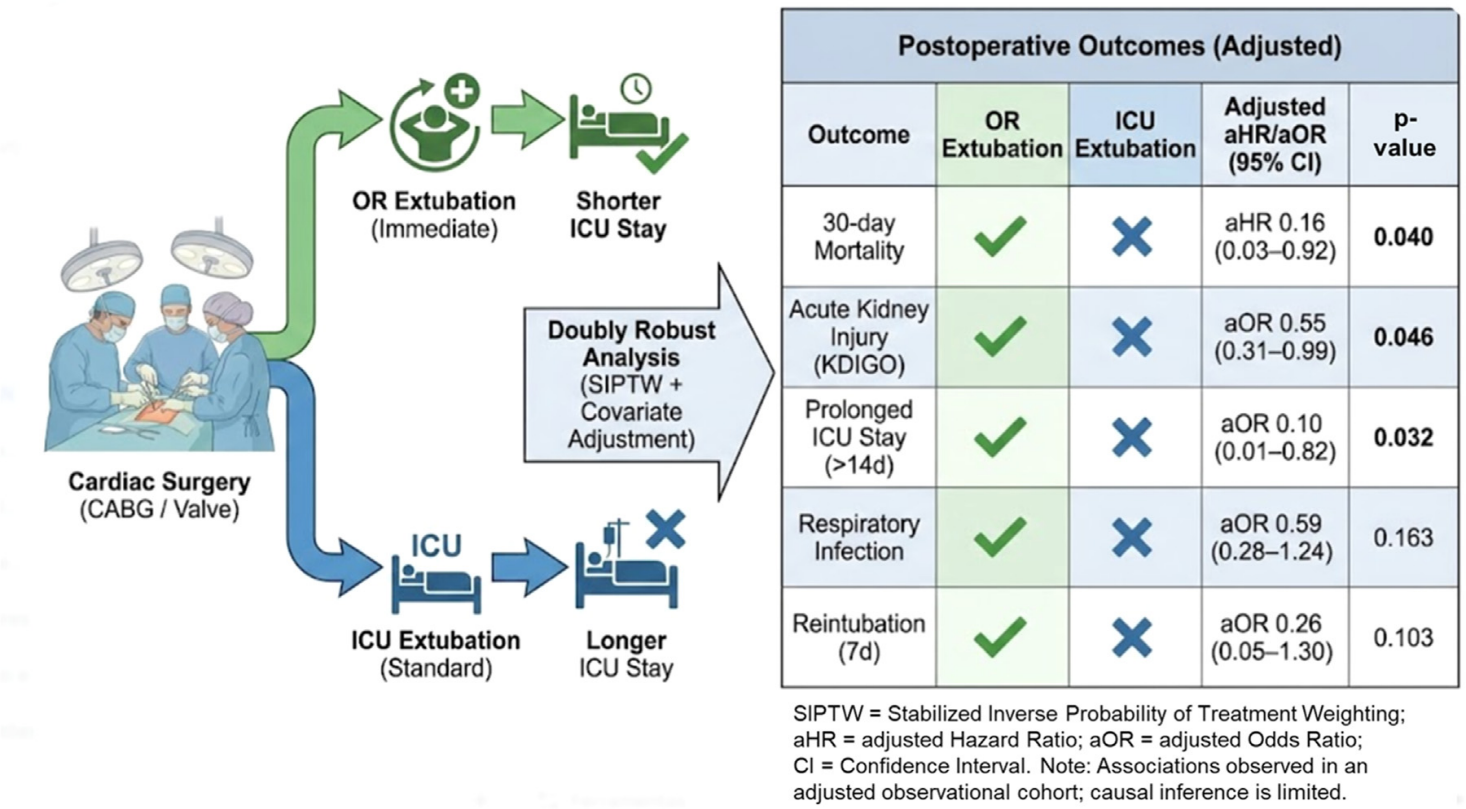
Central Illustration: Associations between operating room extubation on clinical outcomes.

Alt-text: Unlabelled box dummy alt text

## Conflicts of interest

The authors declare no conflicts of interest.

## Appendix Supplementary materials

Image, application 1

## Footnotes

Supplementary material associated with this article can be found in the online version at doi:10.1016/j.bjane.2026.844761.
