# Supplementary material for: Clinical impact of extubation in the operating room after cardiac surgery: a retrospective analysis of a prospective registry
Source: Braz J Anesthesiol. 2026 Apr 24;76(4):844761. doi: 10.1016/j.bjane.2026.844761 (PMC13213226; doi:10.1016/j.bjane.2026.844761)
Supplement: Supplementary file 1 [file mmc1.docx]

**BJAN-D-25-00488_Supplementary Material**

**Supplemental Tables**

**Table S1** Assessment of covariate balance before and after propensity score weighting.

| **Covariate** | **Unadjusted SMD** | **Weighted SMD (SIPTW)** | **Balance Status** |
| --- | --- | --- | --- |
| Demographics |  |  |  |
| Age | -0.245 | -0.197 | Residual Imbalance^a^ |
| Male Sex | -0.119 | -0.034 | Balanced |
| Body Mass Index (BMI) | -0.051 | 0.012 | Balanced |
| **Clinical History** |  |  |  |
| Hypertension | -0.192 | 0.023 | Balanced |
| Diabetes Mellitus | -0.031 | 0.023 | Balanced |
| COPD | -0.064 | -0.087 | Balanced |
| Prior Cardiac Surgery | -0.135 | -0.224 | Residual Imbalance |
| **Preoperative Status** |  |  |  |
| Baseline eGFR (Renal Function) | +0.327 | +0.058 | Balanced |
| Left Ventricular Ejection Fraction | +0.115 | -0.028 | Balanced |
| Urgent/Emergent Status | -0.331 | +0.051 | Balanced |
| **Operative Characteristics** |  |  |  |
| Isolated CABG | -0.378 | +0.006 | Balanced |
| On-Pump Surgery | -0.542 | +0.011 | Balanced |
| CPB Duration | -0.352 | -0.084 | Balanced |

Footnotes: SMD, Standardized Mean Difference. An absolute SMD < 0.10 indicates negligible imbalance. SIPTW, Stabilized Inverse Probability of Treatment Weighting.

^a^ Although age and prior cardiac surgery showed residual imbalance (SMD > 0.10) after weighting, these variables were included as covariates in the final Doubly Robust regression models to ensure full adjustment.

Note: Positive values indicate higher means/prevalence in the OR Extubation group; negative values indicate higher means/prevalence in the ICU Extubation group.

**Table S2** Demographic and risk factors of patients undergoing cardiac surgery according to postoperative survival (unadjusted analysis).

|  | **All patients** | **Survivors** | **Non-survivors** | **p-value** |
| --- | --- | --- | --- | --- |
|  | **n = 846** | **n = 796** | **n = 50** |  |
| **Demographics and Hospitalization** |  |  |  |  |
| Age (years) | 62 (55‒69) | 62 (55‒69) | 68 (63‒71) | < 0.001 |
| 18 to < 40 years | 46 (5.4%) | 46 (5.8%) | 0 (0.0%) | 0.104 |
| 40 to < 65 years | 446 (52.7%) | 431 (54.1%) | 15 (30.0%) | 0.001 |
| 65 to < 75 years | 272 (32.2%) | 241 (30.3%) | 31 (62.0%) | < 0.001 |
| ≥ 75 years | 82 (9.7%) | 78 (9.8%) | 4 (8.0%) | 1.000 |
| Male sex | 578 (68.3%) | 548 (68.8%) | 30 (60.0%) | 0.192 |
| **Risk Factors** |  |  |  |  |
| Weight (kg) | 75 (66‒87) | 75 (66‒86) | 77 (64‒95) | 0.276 |
| Height (m) | 1.67 (1.60‒1.73) | 1.67 (1.60‒1.73) | 1.68 (1.60‒1.72) | 0.898 |
| Body Mass Index (kg.m^-2^) | 27 (25‒31) | 27 (25‒30) | 29 (24‒32) | 0.122 |
| Normal weight (18.5 to < 25.0) | 218 (25.8%) | 202 (25.4%) | 16 (32.0%) | 0.299 |
| Underweight (< 18.5) | 15 (1.8%) | 15 (1.9%) | 0 (0.0%) | 1.000 |
| Overweight (25.0 to < 30.0) | 374 (44.2%) | 362 (45.5%) | 12 (24.0%) | 0.003 |
| Obesity (≥ 30.0) | 239 (28.3%) | 217 (27.3%) | 22 (44.0%) | 0.011 |
| Chronic Obstructive Pulmonary Disease | 39 (4.6%) | 34 (4.3%) | 5 (10.0%) | 0.073 |
| Hypertension | 623 (73.6%) | 583 (73.2%) | 40 (80.0%) | 0.293 |
| Diabetes mellitus | 298 (35.2%) | 280 (35.2%) | 18 (36.0%) | 0.906 |

Footnotes: Values are presented as n (%) for categorical data or as median (IQR) for continuous variables.

**Table S3** Renal and ventricular function, and duration of extracorporeal circulation of patients undergoing cardiac surgery according to postoperative survival (unadjusted analysis).

|  | **All patients** | **Survivors** | **Non-survivors** | **p-value** |
| --- | --- | --- | --- | --- |
|  | **n = 846** | **n = 796** | **n = 50** |  |
| **Laboratory Tests** |  |  |  |  |
| **Renal Function** |  |  |  |  |
| Baseline SCr (mg.dL^-1^) | 1.10 (0.90‒1.34) | 1.10 (0.90‒1.30) | 1.32 (1.10‒2.53) | < 0.001 |
| Elevated baseline SCr | 336 (39.7%) | 301 (37.8%) | 35 (70.0%) | < 0.001 |
| End-stage renal disease | 16 (1.9%) | 11 (1.4%) | 5 (10.0%) | 0.002 |
| Baseline e-GFR (CKD-EPI 2021; mL.min^-1^/1.73 m^2^) | 70 (54‒87) | 71 (56‒88) | 50 (24‒67) | < 0.001 |
| Stage 1/2 (≥ 60) | 568 (67.1%) | 550 (69.1%) | 18 (36.0%) | < 0.001 |
| Stage 3A/3B (30 to < 60) | 233 (27.5%) | 218 (27.4%) | 15 (30.0%) | 0.688 |
| Stage 4/5 (< 30) | 45 (5.3%) | 28 (3.5%) | 17 (34.0%) | < 0.001 |
| **Left Ventricular Ejection Fraction** |  |  |  |  |
| > 50% | 632 (74.7%) | 599 (75.3%) | 33 (66.0%) | 0.144 |
| 31% to 50% | 164 (19.4%) | 151 (19.0%) | 13 (26.0%) | 0.223 |
| ≤ 30% | 50 (5.9%) | 46 (5.8%) | 4 (8.0%) | 0.530 |
| On-pump surgery | 826 (97.6%) | 778 (97.7%) | 48 (96.0%) | 0.333 |
| Duration of CPB (minutes) | 88 (75‒102) | 87 (74‒102) | 93 (78‒116) | 0.054 |
| < 90 | 436 (52.8%) | 416 (53.4%) | 20 (42.6%) | 0.148 |
| 90 to < 120 | 300 (36.3%) | 284 (36.5%) | 16 (34.0%) | 0.738 |
| ≥ 120 | 90 (10.9%) | 79 (10.1%) | 11 (23.4%) | 0.005 |

Footnotes: Values are presented as n (%) for categorical data or as median (IQR) for continuous variables.

SCr, Serum Creatinine; eGFR, Estimated Glomerular Filtration Rate (CKD-EPI); CPB, Cardiopulmonary Bypass.

**Table S4** Patients undergoing coronary artery bypass grafting according to postoperative survival (unadjusted analysis).

|  | **All patients** | **Survivors** | **Non-survivors** | **p-value** |
| --- | --- | --- | --- | --- |
|  | **n = 846** | **n = 796** | **n = 50** |  |
| **Coronary artery bypass grafting** | 452 (53.4%) | 433 (54.4%) | 19 (38.0%) | 0.024 |
| Preoperative Status of CAD |  |  |  |  |
| Chronic stable angina | 260 (57.5%) | 251 (58.0%) | 9 (47.4%) | 0.360 |
| Acute coronary syndrome | 192 (42.5%) | 182 (42.0%) | 10 (52.6%) | 0.360 |
| Unstable angina | 54 (11.9%) | 52 (12.0%) | 2 (10.5%) | 1.000 |
| Non-ST-segment elevation myocardial infarction | 114 (25.2%) | 107 (24.7%) | 7 (36.8%) | 0.279 |
| ST-segment elevation myocardial infarction | 24 (5.3%) | 23 (5.3%) | 1 (5.3%) | 1.000 |
| Left main coronary artery disease (> 50%) | 162 (35.8%) | 153 (35.3%) | 9 (47.4%) | 0.284 |
| Tri-vessel coronary artery disease | 337 (74.6%) | 323 (74.6%) | 14 (73.7%) | 1.000 |
| **Cardiac Assistance Devices** |  |  |  |  |
| Intra-aortic balloon pump | 91 (20.2%) | 84 (19.4%) | 7 (36.8%) | 0.079 |

Footnotes: Values are presented as n (%) for categorical data or as median (IQR) for continuous variables.

CAD, Coronary Artery Disease.

**Table S5** Patients undergoing valve surgery according to postoperative survival (unadjusted analysis).

|  | **All patients** | **Survivors** | **Non-survivors** | **p-value** |
| --- | --- | --- | --- | --- |
|  | **n = 846** | **n = 796** | **n = 50** |  |
| **Valve surgery** | 394 (46.6%) | 363 (45.6%) | 31 (62.0%) | 0.024 |
| Mitral valve repair | 29 (7.4%) | 29 (8.0%) | 0 (0.0%) | 0.151 |
| Mitral valve replacement | 176 (44.7%) | 160 (44.1%) | 16 (51.6%) | 0.418 |
| Aortic valve replacement | 213 (54.1%) | 196 (54.0%) | 17 (54.8%) | 0.928 |
| Tricuspid valve repair or replacement | 85 (21.5%) | 78 (21.4%) | 7 (22.6%) | 0.881 |
| Multiple valve surgery | 98 (24.8%) | 91 (25.0%) | 7 (22.6%) | 0.765 |

Footnotes: Values are presented as n (%) for categorical data or as median (IQR) for continuous variables.

**Table S6** Clinical complications and ICU stay of patients undergoing cardiac surgery according to postoperative survival (unadjusted analysis).

|  | **Overall** | **Survivor** | **Non Survivor** | **p-value** |
| --- | --- | --- | --- | --- |
|  | **n = 846** | **n = 796** | **n = 50** |  |
| **Postoperative outcomes** |  |  |  |  |
| Acute kidney injury (KDIGO)^a^ | 446 (52.7) | 407 (51.1) | 39 (78.0) | < 0.001 |
| KDIGO 1 | 373 (44.1) | 355 (44.6) | 18 (36.0) | 0.235 |
| KDIGO 2 | 36 (4.3) | 28 (3.5) | 8 (16.0) | 0.001 |
| KDIGO 3 | 37 (4.4) | 24 (3.0) | 13 (26.0) | < 0.001 |
| RRT up to 7 days | 14 (1.7) | 8 (1.0) | 6 (12.0) | < 0.001 |
| Reoperation for bleeding/ Tamponade | 24 (2.8) | 20 (2.5) | 4 (8.0) | 0.048 |
| Acute atrial fibrillation | 137 (16.2) | 126 (15.8) | 11 (22.0) | 0.251 |
| Respiratory infection | 188 (22.2) | 160 (20.1) | 28 (56.0) | < 0.001 |
| Tracheal reintubation up to 7 days | 32 (3.8) | 19 (2.4) | 13 (26.0) | < 0.001 |
| Deep sternal wound infection | 37 (4.4) | 33 (4.1) | 4 (8.0) | 0.269 |
| Type 1 neurological injury | 36 (4.3) | 28 (3.5) | 8 (16.0) | 0.001 |
| **Discharge** |  |  |  |  |
| CS-ICU readmission | 48 (5.7) | 40 (5.0) | 8 (16.0) | 0.005 |
| CS-ICU LOS up to 30 days | 4 (3 - 6) | 4 (3 - 5) | 5 (2 - 12) | 0.299 |
| Long LOS (> 14 days) | 48 (5.7) | 39 (4.9) | 9 (18.0) | 0.001 |

Footnotes: Values are presented as n (%) for categorical variables and median (IQR) for continuous variables. p-values calculated using Chi-Square test, Fisher’s exact test, or Mann-Whitney *U*-test, as appropriate. These represent unadjusted comparisons.

^a^ Acute Kidney Injury defined according to KDIGO (Kidney Disease: Improving Global Outcomes definition and staging) criteria.

**Table S7** Unadjusted Clinical Complications, ICU Stay, and Mortality in Patients Undergoing Isolated Coronary Artery Bypass Grafting (CABG) According to Extubation Location.

|  | **Overall** | **OR extubation** | **ICU extubation** | **p-value** |
| --- | --- | --- | --- | --- |
|  | **n = 452** | **n = 43** | **n = 409** |  |
| **Postoperative outcomes** |  |  |  |  |
| KDIGO | 229 (50.7) | 16 (37.2) | 213 (52.1) | 0.064 |
| KDIGO 1 | 192 (42.5) | 15 (34.9) | 177 (43.3) | 0.290 |
| KDIGO 2 | 23 (5.1) | 1 (2.3) | 22 (5.4) | 0.713 |
| KDIGO 3 | 14 (3.1) | 0 (0.0) | 14 (3.4) | 0.381 |
| RRT up to 7 days | 5 (1.1) | 0 (0.0) | 5 (1.2) | 1.000 |
| Reoperation for bleeding/tamponade | 12 (2.7) | 0 (0.0) | 12 (2.9) | 0.615 |
| Acute atrial fibrillation | 66 (14.6) | 5 (11.6) | 61 (14.9) | 0.562 |
| Respiratory infection | 114 (25.2) | 8 (18.6) | 106 (25.9) | 0.294 |
| Tracheal reintubation up to 7 days | 15 (3.3) | 0 (0.0) | 15 (3.7) | 0.381 |
| Prolonged pulmonary ventilation (> 24 hours) | 29 (6.4) | 0 (0.0) | 29 (7.1) | 0.096 |
| Operating room extubation | 43 (9.5) | 43 (100.0) | 0 (0.0) | ‒ |
| Deep sternal wound infection | 27 (6.0) | 2 (4.7) | 25 (6.1) | 1.000 |
| Type-1 neurological injury | 8 (1.8) | 0 (0.0) | 8 (2.0) | 1.000 |
| **Discharge and mortality** |  |  |  |  |
| CS-ICU readmission | 22 (4.9) | 2 (4.7) | 20 (4.9) | 1.000 |
| CS-ICU LOS up to 30 days | 4 (3‒5) | 3 (2‒4) | 4 (3‒5) | 0.009 |
| Long LOS (> 14 days) | 22 (4.9) | 1 (2.3) | 21 (5.1) | 0.710 |
| **30-day mortality** | 19 (4.2) | 0 (0.0) | 19 (4.6) | 0.239 |

Footnotes: Values are presented as n (%) for categorical variables and median (IQR) for continuous variables. The p-values were calculated using Chi-Square test, Fisher’s exact test, or Mann-Whitney *U*-test, as appropriate. These represent unadjusted comparisons.

^a^ Acute Kidney Injury defined according to KDIGO (Kidney Disease: Improving Global Outcomes definition and staging) criteria.

**Table S8** Unadjusted clinical complications, ICU stay, and mortality in patients undergoing Heart Valve Surgery (HVS) according to extubation location.

|  | **Overall** | **OR extubation** | **ICU extubation** | **p-value** |
| --- | --- | --- | --- | --- |
|  | **n = 394** | **n = 72** | **n = 322** |  |
| **Postoperative outcomes** |  |  |  |  |
| KDIGO | 217 (55.1) | 30 (41.7) | 187 (58.1) | 0.011 |
| KDIGO 1 | 181 (45.9) | 28 (38.9) | 153 (47.5) | 0.184 |
| KDIGO 2 | 13 (3.3) | 2 (2.8) | 11 (3.4) | 1.000 |
| KDIGO 3 | 23 (5.8) | 0 (0.0) | 23 (7.1) | 0.012 |
| RRT up to 7 days | 9 (2.3) | 0 (0.0) | 9 (2.8) | 0.375 |
| Reoperation for bleeding/tamponade | 12 (3.0) | 1 (1.4) | 11 (3.4) | 0.703 |
| Acute atrial fibrillation | 71 (18.0) | 12 (16.7) | 59 (18.3) | 0.741 |
| Respiratory infection | 74 (18.8) | 6 (8.3) | 68 (21.1) | 0.012 |
| Tracheal reintubation up to 7 days | 17 (4.3) | 2 (2.8) | 15 (4.7) | 0.749 |
| Prolonged pulmonary ventilation (> 24 hours) | 48 (12.2) | 1 (1.4) | 47 (14.6) | 0.002 |
| Operating room extubation | 72 (18.3) | 72 (100.0) | 0 (0.0) | ‒ |
| Deep sternal wound infection | 10 (2.5) | 1 (1.4) | 9 (2.8) | 0.697 |
| Type 1 neurological injury | 28 (7.1) | 4 (5.6) | 24 (7.5) | 0.571 |
| **Discharge and mortality** |  |  |  |  |
| CS-ICU readmission | 26 (6.6) | 2 (2.8) | 24 (7.5) | 0.193 |
| CS-ICU LOS up to 30 days | 4 (3‒6) | 3 (2‒4) | 4 (3‒6) | < 0.001 |
| Long LOS (> 14 days) | 26 (6.6) | 0 (0.0) | 26 (8.1) | 0.007 |
| **30-day mortality** | 31 (7.9) | 2 (2.8) | 29 (9.0) | 0.076 |

Footnotes: Values are presented as n (%) for categorical variables and median (IQR) for continuous variables. The p-values were calculated using Chi-Square test, Fisher’s exact test, or Mann-Whitney *U*-test, as appropriate. These represent unadjusted comparisons.

^a^ Acute Kidney Injury defined according to KDIGO (Kidney Disease: Improving Global Outcomes definition and staging) criteria.
